# Arylamine N-acetyl Transferase (NAT) in the blue secretion of *Telescopium telescopium*: xenobiotic metabolizing enzyme as a biomarker for detection of environmental pollution

**DOI:** 10.1186/2193-1801-3-666

**Published:** 2014-11-11

**Authors:** Bapi Gorain, Sumon Chakraborty, Murari Mohan Pal, Ratul Sarkar, Samir Kumar Samanta, Sanmoy Karmakar, Tuhinadri Sen

**Affiliations:** Department of Pharmaceutical Technology, Jadavpur University, Kolkata, 700032 India; School of Natural Product Studies, Jadavpur University, Kolkata, 700032 India; Calcutta Institute of Pharmaceutical Technology and AHS, Banitabla, Ulluberia, Howrah 711316 India

## Abstract

*Telescopium telescopium*, a marine mollusc collected from Sundarban mangrove, belongs to the largest mollusca phylum in the world and exudes a blue secretion when stimulated mechanically. The blue secretion was found to metabolize (preferentially) para-amino benzoic acid, a substrate for N-acetyl transferase (NAT), thereby indicating acetyl transferase like activity of the secretion. Attempts were also made to characterise bioactive fraction of the blue secretion and to further use this as a biomarker for monitoring of marine pollution. NAT like enzyme from marine mollusc is a potential candidate for detoxification of different harmful chemicals. A partially purified extract of blue secretion was obtained by fractional precipitation with (NH_4_)_2_SO_4_. From different fractions obtained by precipitation, the 0–30% fraction (30S) displayed NAT like activity (using para amino benzoic acid as a substrate with para nitrophenyl phosphate or acetyl coenzyme A as acetyl group donors). Maximum NAT like enzyme activity was attained at 25°C and at a pH of 6. The enzyme activity was found to be inhibited by 5 mM phenyl methyl sulfonyl fluoride. The divalent metal ions reduced NAT like activity of 30S. Moreover, Cu^2+^ and Zn^2+^ (at concentration of 1 mM) completely inhibited NAT activity. The thermal stability and bench-top stability studies were performed and it was found that the enzyme was stable at room temperature for more than 24 hours. Results from the present study further indicate that heavy metal content in blue secretion gradually decreased from pre-monsoon to post-monsoon season, which also corresponded to the change in NAT like activity. Therefore, this article stresses the importance of biomarker research for monitoring pollution.

## Background

**Figure 1:**
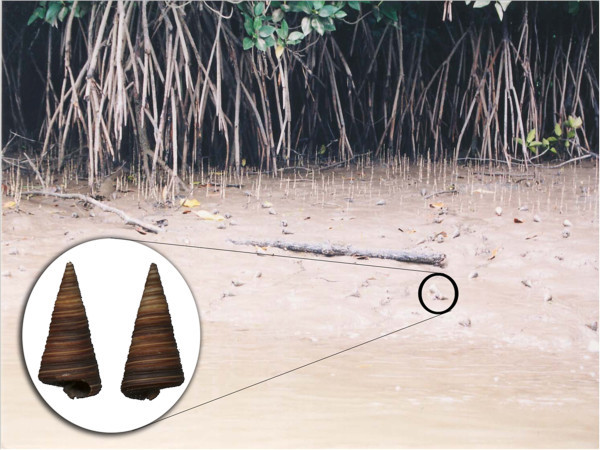
**Mollusk** ***Telescopium telescopium*** **and its habitat.**

*Telescopium telescopium* (Linnaeus, 1758) (mud whelk or mangrove snail), a mangrove gastropod mollusc, is the most dominant molluscan species of Sundarban mangrove (Figure 1). In terms of total number of species, mollusca are numerically second largest phyla after arthropoda, among the invertebrates found in Sundarban ecosystem. Although, this species is widely consumed as food in other parts of the world (Swadling 1977), such practice is uncommon in Sundarbans.

The mollusc is found abundantly in the coastline of India and resides mainly in the estuarine environment in the basin of different rivers. It is well known that this particular mollusc species secretes a blue coloured viscous liquid when disturbed by any external mechanical stimuli. The capability of surviving in the intertidal zone may make them an attractive subject for exploring the impact of environmental pollutants.

According to the available scientific information, organisms could serve as biomonitors of heavy metals and could be used effectively in ecotoxicological assessment, offering a scope for establishing a direct correlation with metal contamination. The marine bivalves have been used since 1970s as sentinel species for pollution monitoring because of their capability to bioaccumulate and magnify many contaminants (Sarkar et al. 2008; Zuykov et al. 2013).

Reports mentioning the use of enzymes like acetyl cholinesterase (from marine organisms), as a marker for biomonitoring of marine pollution (Gaitonde et al. 2006; Pfeifer et al. 2005; Sturm et al. 1999; Escartin and Porte 1997), are now available in the scientific literature. *Telescopium telescopium* has also been used for biomonitoring of metal contaminants for assessing the pollution level at Dumai (coastal region) in Indonesia (Yap and Noorhaidah 2011; Amin et al. 2005). Studies with *Ruditapes decussatus* and *Mytilus galloprovincialis* have revealed the impact of seasonal changes on acetyl cholinesterase activity which was found to vary with seasons, as well as with heavy metal concentration (Dellali et al. 2001).

Xenobiotic metabolizing enzymes are known to protect organisms from the environmental toxicants and have been conserved indifferent life forms. N-acetyl transferase (NAT) isoforms have been found to play a significant role in metabolic process (phase II metabolism of drug and xenobiotics). In this metabolic phase, N-acetylation of drugs and carcinogens often lead to either bioactivation or detoxification of these molecules. Such N-acetylation is known to occur in presence of an acetyl group donor (like acetyl coenzyme A). However, genetic polymorphism in NAT may lead to enhanced susceptibility of individuals to toxic effects of drugs and carcinogens. NAT has been known to play role in xenobiotics detoxification, particularly in the prokaryotes, thereby protecting the hosts from extreme environmental conditions (Vagena et al. 2008). Genetic surveys for understanding the distribution of polymorphic NAT homologues, across different taxonomic groups, has revealed partial NAT-like ESTs in *Lottia gigantean* (a mollusc) and in arthropods *Litopenaeus vannamei* (Glenn et al*.*^2010^). However, there is a dearth of information regarding the utilisation of xenobiotic biotransforming enzymes like arylamine N-acetyl transferase (NAT), for biomonitoring of the environment. Based on polymorphism, intrinsic stabilities, and as well as substrate specificity, NAT can be classified as (i) NAT-1 (arylamine-NAT), utilizing only arylamine as substrate, like PABA and (ii) NAT-2 (mixed arylamine/arylalkylamine-NAT), that utilizes arylamine and aryl-alkylamine, as its substrate (Gaudet et al. 1993; Sim et al. 2008). Based on substrate utilization, human NAT-1 has been found to be homologus to rabbit NAT-1 and mouse NAT-2 (Sim et al. 2008).

Survey of scientific literature reveals very little information regarding the biochemical and pharmacological properties of *Telescopium telescopium*. Accordingly, investigations were taken up in our laboratory for evaluation of pharmacological and biochemical properties of *Telescopium telescopium* (tissue extract and blue secretion). Earlier studies revealed neuro-pharmacological (Samanta et al. 2008a) haemolytic, pro-inflammatory and hypotensive properties (Samanta et al. 2008b) of tissue extract of *Telescopium telescopium.* The pharmacological and antimicrobial properties of spermathecal gland of *Telescopium telescopium* has also been reported (Datta et al. 2010; Pakrashi et al. 1992; Pakrashi et al. 2001).

In the present investigation, an attempt has been made to explore biochemical properties of blue secretion of *Telescopium telescopium*, with particular references to biomonitoring of Sundarban mangroves.

## Results

The present study was an attempt to detect the presence of a biomarker (enzyme) from blue secretion of *Telescopium telescopium.* Biochemical characterization of biomarker was also performed. The present study also focuses on possible correlation of biomarker with different heavy metals (detected in mangrove environment).

### Protein fractionation and determination of NAT activity

**Figure 2:**
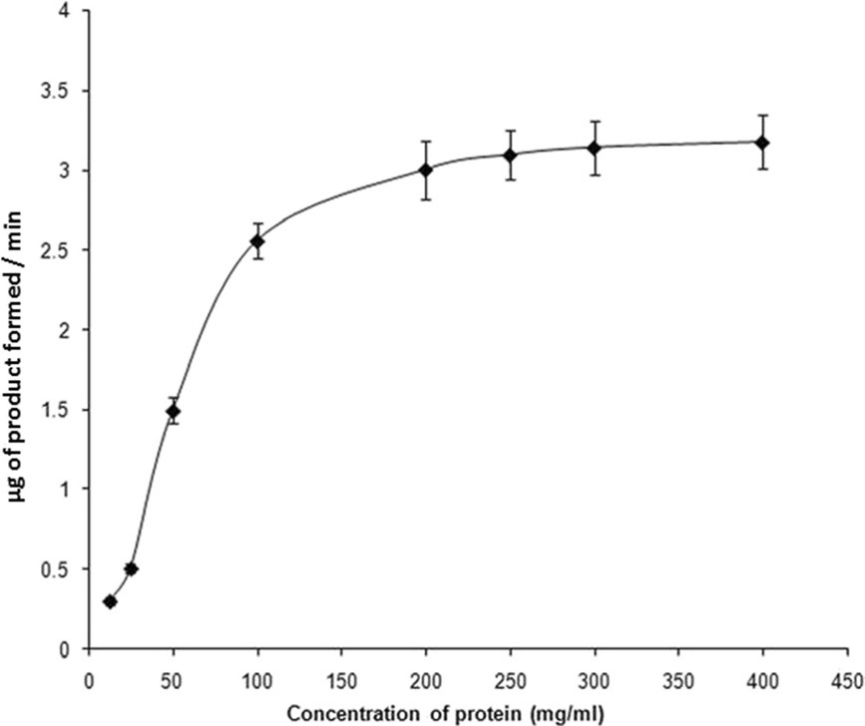
**NAT like enzyme activity of the bioactive (30S) fraction of the mollusk secretion at different concentration of protein in the reaction mixture.** Results shown are mean ± SE (n = 3).

NAT activity was not detected with 0-60% and 60-80% (NH4)2SO4 precipitated fractions. The 0-30% (NH4)2SO4 fraction of the blue secretion demonstrated significant (*p* <0.05) NAT activity. Therefore further biochemical studies were attempted with bioactive hereafter, referred to as 30 S (0-30% (NH4)2SO4 fraction). From our findings, NAT like enzyme activity was found to be concentration dependent (Figure 2).

### Hydrolysis of acetyl coenzyme A

**Figure 3:**
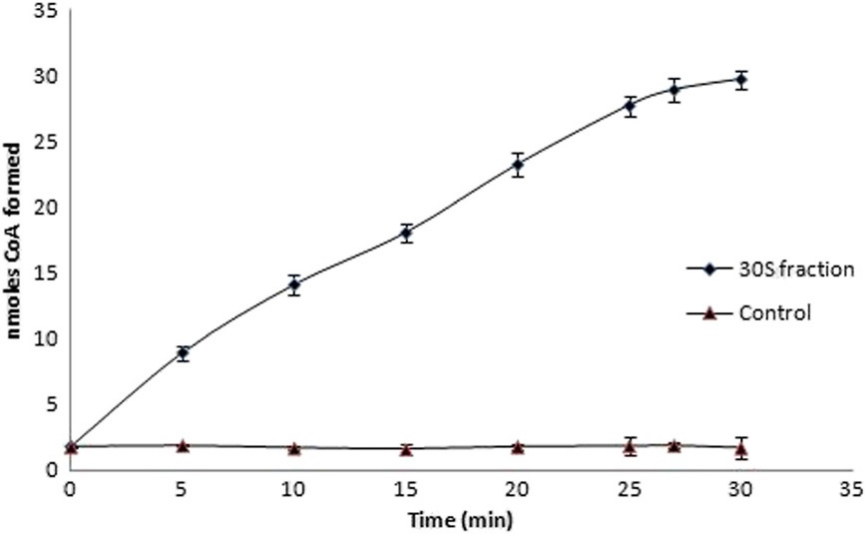
**The use of DTNB to determine the rate of hydrolysis of acetyl coenzyme A in the presence of PABA and 30S fraction.** The quantity of CoA was determined with DTNB. Results shown are mean ± SE (n = 3).

The NAT catalysed acetylation of arylamines results in hydrolysis of acetyl Coenzyme A (AcCoA) to give free Coenzyme A (CoA). 5, 5′-dithio-bis (2-nitrobenzoic acid (DTNB) reacts with free thiol groups in solution to produce thio-nitrobenzoate (TNB). Here, PABA, 30S fraction (from mollusk) and AcCoA were incubated for varying time periods, in a 96-well plate and then DTNB was added. TNB was detected spectrophotometrically and the amount of TNB formed, was found to be dependent on the time of incubation of NAT/substrate mixture (Figure 3). Hydrolysis of AcCoA was not observed when incubated in absence of substrate or enzyme.

### Effect of seasonal variation on NAT activity

**Figure 4:**
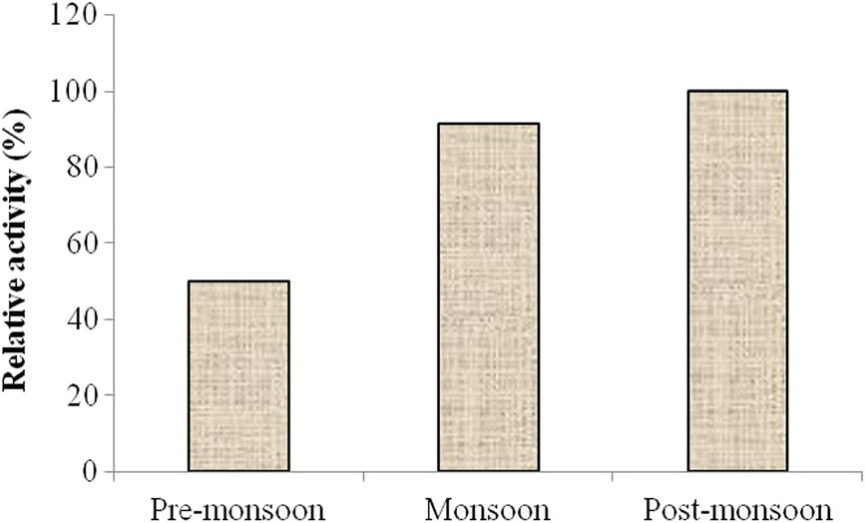
**Alteration of NAT like enzyme activity of the bioactive (30S) fraction of the mollusk secretion with a fixed dose of 200** **μg protein equivalent with respect to seasonal change.**

The result clearly demonstrates presence of NAT like activity in 30S fraction (Figure 4), particularly during monsoon (August) and post-monsoon periods (November), whereas, the enzyme activity was found to be reduced during pre-monsoon (April).

### pH Optima

**Figure 5:**
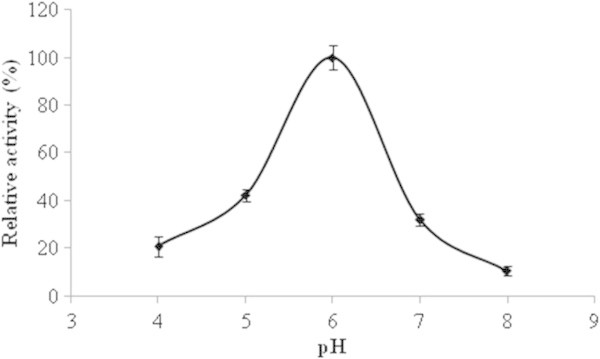
**pH dependent change of NAT like enzyme activity of the bioactive (30S) fraction of the mollusk secretion with a fixed dose of 200** **μg protein equivalent.** Results shown are mean ± SE (n = 3).

Maximal NAT like activity of 30 S fraction was observed at pH 6.0 (20 mM Tris–HCl), and the activity decreased with change in pH of reaction medium (Figure 5). In our study, pH for optimum enzyme activity was found to be different when compared to activity of NAT enzymes isolated from chicken liver (pH 5.0) (Deguchi et al. 1988), from bacteria *Aeromonas hydrophilia* (pH 7.0) (Chung 1998), mammalian (mice) (pH 7.0) (Mattano et al. 1989), or (*Musca domestica*) (pH 7.2) (Whitaker and Goosey 1993).

### Effect of incubation temperature

**Figure 6:**
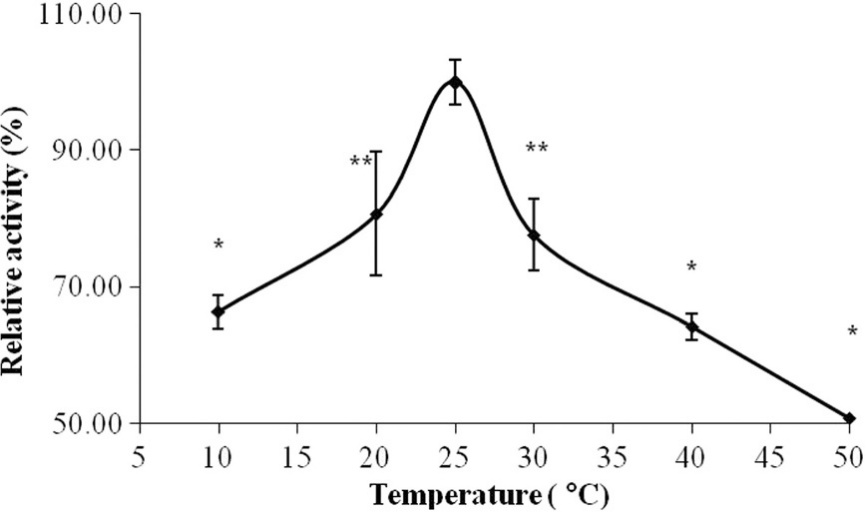
**Temperature dependent change of NAT like enzyme activity of the bioactive (30S) fraction of the mollusk secretion.** Results shown are mean ± SE (n = 3).

The optimum temperature was found to be 25°C, and activity decreased on increasing incubation temperature (Figure 6). NAT enzyme obtained from different sources has been found to display diverse temperature optima.

### Effect of divalent cations and inhibitors

Metal ions such as Zn^+2^ and Cu^+2,^ completely abolished the activity, while Mg^+2^, Mn^+2^, reduced the activity by 80–90% (Table 1). Calcium (Ca^+2^) at concentrations of 1 mM reduced activity to 66%, whereas at 5 mM concentration abolished the enzyme activity completely. These findings were found to be similar to NAT enzyme activity of bacterial origin (Chung 1998).

**Table 1:** **Effect of divalent cations on NAT like enzyme activity (n = 3) of the bioactive (30S) fraction of the mollusk secretion (200** μ**g protein equivalent)**

Divalent ions	Concentration	% retention of activity*	***p*** Value ^**^
Cu^+2^	1 mM	0	< 0.001
	5 mM	0	< 0.001
Mg^+2^	1 mM	12.03 ± 0.97	< 0.001
	5 mM	7.11 ± 0.60	< 0.001
Mn^+2^	1 mM	10.07 ± 0.98	< 0.001
	5 mM	6.97 ± 0.74	< 0.001
Ca^+2^	1 mM	34.01 ± 2.07	< 0.001
	5 mM	0.00	< 0.001
Zn^+2^	1 mM	0.00	< 0.001
	5 mM	0.00	< 0.001

^*^Data are presented as the mean ± standard deviation (n = 3); ^**^Statistical analysis was performed by comparing with control group.

NAT like activity was significantly reduced in the presence of PMSF and EDTA, whereas other compounds produced negligible effects on NAT like activity of 30S sample (Table 2).

**Table 2:** **Effect of inhibitors on NAT like enzyme activity (n = 3) of the bioactive (30S) fraction of the mollusk secretion (200** μ**g protein equivalent)**

Inhibitors	% inhibition of activity ^*^	p Value ^**^
Hexamethonium	9.88 ± 1.01	< 0.001
Decamethnium	13.6 ± 1.19	< 0.001
PMSF	67.9 ± 3.90	< 0.001
EDTA	39.5 ± 2.91	< 0.001

^*^Data are presented as the mean ± standard deviation (n = 3); ^**^Statistical analysis was performed by comparing with control group.

### Bench-top stability studies

**Figure 7:**
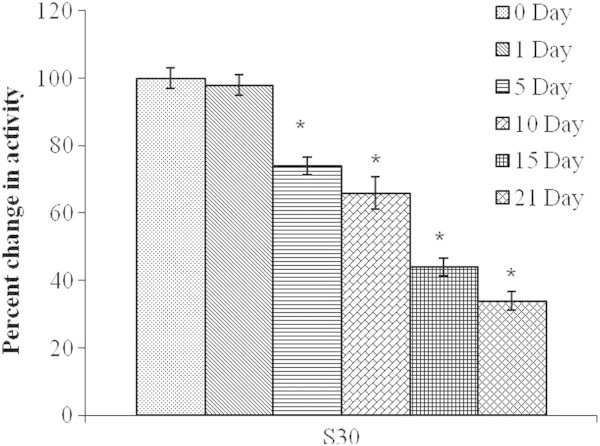
**Bench top stability (at room temperature), of the NAT like enzyme activity of the bioactive (30S) fraction of the mollusk secretion.** Results shown are mean ± SE (n = 3).

The purified enzyme was kept at room temperature (25 ± 2°C) for 21 days. The enzyme activity of fraction (30S) decreased gradually and 34% of NAT like enzyme activity was retained on the 21^st^ day (Figure 7).

### Studies on substrate specificity

**Figure 8:**
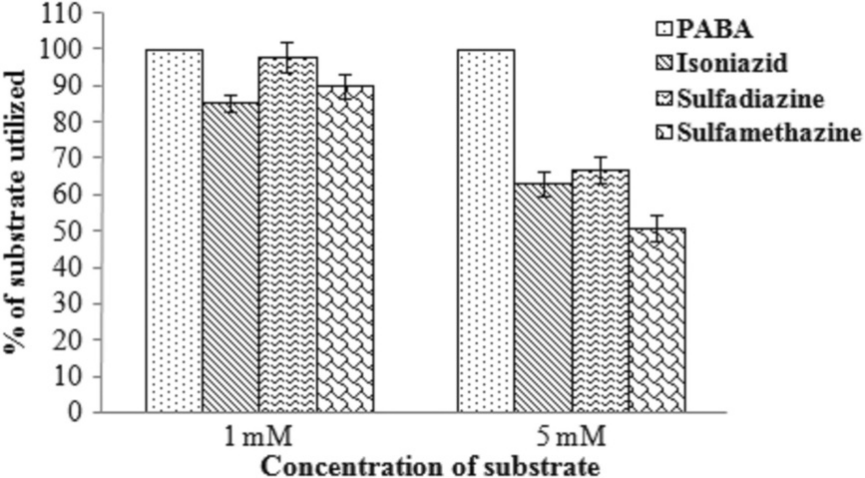
**Substrate specificity of the NAT like enzyme of the bioactive (30S) fraction of the mollusk secretion.** Results shown are mean ± SE (n = 3).

The substrate specificity was evaluated (for NAT like enzyme activity) using different arylamine substrates (PABA, isoniazide, sulfadiazine, and sulfamethazine); maximum activity was observed in the presence of PABA and the activity was found to be concentration dependent (Figure 8).

### Analysis of heavy metal ions in the blue secretion

As evident is from Table 3, the heavy metal content in the secretion was found to be low during the monsoon and post monsoon period. The heavy metal concentration obtained during monsoon period (Hg: 17.52 ± 1.49 μg/g, As: 5.51 ± 0.241 μg/g, Pb: 0.84 ± 0.042 μg/g) was found to decrease during pre-monsoon to monsoon and monsoon to post monsoon (Table 3). Moreover, NAT like activity of 30S fraction was also found to be altered with change in concentrations of heavy metal (Figure 2).

**Table 3:** **Results of the heavy metal content in the blue secretion of the mollusk** ***Telescopium telescopium*** **(on seasonal basis)**

Season	Mercury (μg/g)	Arsenic (μg/g)	Lead (μg/g)
Pre-monsoon	17.4 ± 0.819	5.51 ± 0.624	0.84 ± 0.081
Monsoon	11.3 ± 0.745	3.26 ± 0.256	BDL
Post-monsoon	7.16 ± 0.459	0.08 ± 0.008	BDL

Data are presented as the mean ± standard deviation (n = 3).

BDL – below detectable limit.

## Discussion

NAT enzymes are polymorphic xenobiotic metabolizing enzymes, found in almost all living beings, except plants (Sabbagh et al. 2013). Apart from human beings, presence of NAT has been confirmed in many species including rabbits, birds, frogs, nematodes, fish, and bacteria (Hui et al. 2004). It plays a major role in detoxification of carcinogens and various arylamine drugs by bio-transforming lipophilic xenobiotics to its hydrophilic metabolites. Interestingly, survival of *Ralstonia metallidurams* and *Hynobius cheynensis,* under high metal and salt concentrations, has also been correlated to metabolic activity of NAT (Vagena et al. 2008).

The estuarine regions of Sundarbans, despite being a biodiversity hotspot and a world heritage site, is exposed to pollutants (swage and industrial) from the Ganges, Damodar (upstream steel industries), Rupnarayan, and Haldi rivers (Haldia port complex and petrochemical industries). Earlier studies with bivalve mollusks (from the Sundarban mangroves), indicate their ability to bio-accumulate metals (beyond safety standards specified by FAO) (Sarkar et al. 2008). It may also be important to mention that bivalves have high ability for bio-accumulation, therefore used for bio-monitoring, even in situation where the chemicals are present below the detectable limit (Zuykov et al. 2013).

Therefore, on the basis of our observations, it can be suggested that NAT like activity of blue secretion (30S fraction) showing substrate specificity to (PABA), to be similar to that of human NAT-1 (Sim et al. 2008; Kawamura et al. 2005; Blum et al. 1990). However, this finding needs further corroboration from N-terminal amino acid sequencing and other physicochemical characterizations. Considering the ability of the NAT’s to act as either slow or fast acetylators, it may be mentioned that previous study with NAT (purified from New Zealand white rabbits) indicated differential N-acetyltransferase activity towards substrates like sulphamethazine or p-aminobenzoic acid (PABA) (Hearse and Weber 1973). Moreover, such variation was found to be both individual and tissue specific, thereby indicating the possibility of existence of two isoforms, in varying proportion (Hearse and Weber 1973). Similar difference have also been observed with NAT from *Klebsiella pneumonia*, where NAT activity on 2-aminoflurane (substrate for NAT1 and NAT2) was found to higher as compared to PABA, which is known to be a specific substrate for NAT1 (Hui et al. 2004). In the present study, assays were performed with p-nitrophenyl acetate and AcCoA, serving as acetyl group donors and PABA as the acceptor. The 30S fraction of blue secretion was able to both hydrolyse both p-nitrophenyl acetate and AcCoA (Brooke et al. 2003). Biomarkers are known to be altered depending on the level of environmental contamination. According to available reports, the level of contamination in the estuarine region is known to be high during pre-monsoon, because of the presence of high concentration of heavy metal (discharged untreated effluents from different chemical industries) and simultaneous decrease of water flow in the river(s); whereas during monsoon season, there is heavy influx of fresh water from the Matla river (Sundarban), leading to reduced concentration of heavy metals in the estuarine region (Kumar et al. 2011; Joseph and Srivastava 1993). Therefore, the significant increase in NAT like activity during monsoon and post-monsoon periods may be attributed to decreased level of heavy metal contamination in sampling sites.

Hence, the observed changes in toxic heavy metal content (As, Hg and Pb) in blue secretion of mollusc (*Telescopium telescopium)* may play a major role towards the alteration of N-acetyl transferase (NAT) like activity, during different seasons. In our present study, seasonal alteration of NAT like enzyme activity of blue secretion, may in turn affect metabolism of xenobiotics. Similar studies have been carried out with acetylcholine esterase (Gaitonde et al. 2006; Pfeifer et al. 2005; Sturm et al. 1999; Escartin and Porte 1997) for studying of marine pollution. Similarly, Tsangaris and his team analysed different biomarkers in mussels to assess the effect of various pollutants (Tsangaris et al. 2010).

## Conclusion

The present study revealed some important information regarding the molluscan species inhabiting the Sunderban mangroves. The study was able to reveal the presence of arylamine N-acetyl transferase-type 1 (NAT-1) like enzyme activity in the blue secretion and the present report regarding the presence of NAT activity in *Telescopium telescopium* and its subsequent application in environmental monitoring, is the first of its kind to be reported. Further studies would be attempted to purify and characterize the enzymatic component for obtaining structural information related to active site and substrate specificities. Moreover based on our findings, it may be worthy to suggest that NAT like protein (from mollusk or from other animals species inhabiting the coastal areas) may be explored for bio-monitoring (studying coastal pollution in other regions of the world) and also for biosensor applications.

## Materials and methods

### Chemicals

Bovine serum albumin (BSA), ammonium sulfate, p-amino benzoic acid (PABA), p-nitro phenyl acetate (PNPA), trizma base (tris), iodo acetic acid, hexamethonium bromide, decamethonium bromide and protein estimation kit (Bradford Method) were obtained from Sigma. Phenyl methyl sulfonyl fluoride (USB, Switzerland) and DTT (SRL, India). All other chemicals and reagents were of analytical grade (Merck, India), unless or otherwise mentioned.

### Collection and identification of *Telescopium telescopium*

Live molluscan species *Telescopium telescopium* (around 25), were collected from creeks of the river Matla in Jharkhali (88.36E and 22.57 N), Sundarban (West Bengal, India), at the time of low tide (during April, August and November). The molluscan specimens were immediately transported to the laboratory in clean plastic containers. The specimens were identified and authenticated by the Zoological Survey of India (ZSI) New Alipore, Kolkata. Experiments on molluscan specimens were performed following standard guidelines of animal ethics committee.

### Extraction procedure

#### Blue secretion

The specimens were thoroughly washed and then used for obtaining collecting blue secretion. The intact live mollusks (thoroughly cleaned with distilled water) were subjected to external mechanical stimuli by means of a sharp object. The mollusc under physical stress produced a blue secretion, which was immediately collected in a container. The secretion was centrifuged under cold condition at 5000 rpm for 10–15 min, until a clear supernatant was obtained (crude secretion). Secretions from the mollusks (around 15) were pooled in a sterile container. An aliquot of pooled sample was defatted using dichloromethane (DCM) (Samanta et al. 2008b) for protein fractionation (discussed below).

The protein concentration was determined by the dye- binding method of Bradford ( 1976), using a UV–VIS spectrophotometer (Hitachi U2000). Bovine serum albumin (BSA) was used as a protein standard.

### Protein fractionation (precipitation technique)

Ammonium sulfate [(NH_4_)_2_SO_4_] precipitation is one of the most widely used techniques for fractionation of proteins (Scopes 1982). The DCM cut fraction was carefully mixed with ammonium sulfate to obtain a saturation of 30% and the mixture was allowed to stand for 30–45 min and then centrifuged at 10,000 rpm for 25–30 min. The supernatant was the starting material for next fractionation and the pellet was dissolved in small amount of sodium phosphate buffer (20 mM, pH 7.2 with 1 mM EDTA) and labeled as 30% ammonium sulphate (30S) fraction. Similarly, 60% and 80% ammonium sulfate precipitation were also performed. The fractions were dialyzed (molecular weight cut off 3 kDa) for 24 hours at 0-4°C. The dialyzed fraction was stored at -20°C until further use.

### Determination of NAT like activity

The NAT activity was determined by spectrophotometric assay (in triplicate), according to the method of Wang et al. ( 2005) with slight modifications. The reaction mixture in Tris–HCl buffer (20 mM, pH 6.0 with 1 mM DTT, and 1 mM EDTA) was mixed with PABA (0.5 mM). PNPA dissolved in DMSO (0.8 mM) was used as the acetyl group donor. The reaction rate was determined by monitoring the increase in absorbance at 400 nm (Hitachi U2000 Spectrophotometer). The specific activities were expressed as μmol of product formed per mg of protein/min.

### Hydrolysis of AcCoA

The substrate (300 μM) and the 30S fraction (200 μg/ml) were mixed and pre-incubated (37°C, 5 min) in a 96-well plate; pre-warmed AcCoA (400 mM) was added to start the reaction. After appropriate incubation, colour development was achieved by addition of DTNB (5 mM in 0.1 M Tris–HCl, 6.4 M guanidine–HCl pH 7.3, 25 μL). The absorbance was measured at 405 nm (Multiscan GO microplate reader; Thermo) within 5 min. When a solution of CoA (20 mM Tris–HCl, pH 8.0) is treated with DTNB solution (6.4 M guanidine–HCl, 0.1 M Tris–HCl, pH 7.3) in a 96-well plate made of polystyrene (TNB has an extinction of 3.3 ± 0.1 mmol^-1^ dm^3^ cm^-1^ at 405 nm). Reactions performed without substrate, AcCoA or 30S fraction were used as controls. The amount of CoA produced (in triplicate) was determined from a standard curve (Brooke et al. 2003).

### Effect of seasonal variation on NAT activity

The NAT like activity of fraction (30S), collected during pre-monsoon (May), monsoon (August) and post-monsoon (November) were determined by the same assay procedure (stated above) using 200 μg of protein (in triplicate).

### Effect of pH

The effect of pH on NAT activity (in triplicate) was determined by exposing the test samples to different pH conditions (pH 4.0, 5.0, 6.0, 7.0 and 8.0) for 30 min (Adhikari et al. 2007).

### Effect of incubation temperature

The effect of temperature on NAT activity was determined in triplicate by incubating the test samples at specific temperatures (i.e. 10°C, 20°C, 25°C, 30°C, 40°C and 50°C) for 10 min prior to the commencement of the NAT assay (Adhikari et al. 2007).

### Effect of divalent cations and different inhibitors

Specified concentrations (1 mM and 5 mM) of different divalent cations (viz. Ca^+2^, Mg^+2^, Cu^+2^, Zn^+2^, and Mn^+2^) were pre-incubated form 15 min (Adhikari et al. 2007) with the 30S fraction and then the NAT like activity of the samples were determined in triplicate. To evaluate the effect of inhibitors, the test samples were pre-incubated with hexamethonium bromide, decamethonium bromide, ethylene diamine tetra-acetic acid (EDTA) and phenyl methyl sulfonyl fluoride (PMSF) solutions (5 mM), for 15 min. Thereafter NAT activity of the pre-incubated samples was determined at 400 nm (Hitachi U2000 Spectrophotometer).

### Bench-top stability

In this experiment, the test samples were kept at room temperature for a period of three weeks and assayed for NAT activity (described above) on the 0^th^, 1^st^, 5^th^, 10^th^, 15^th^ and 21^st^ day (in triplicate).

### Evaluation of substrate specificity

The blue secretion was incubated with different substrates (PABA, isoniazide, sulfadiazine and sulfamethazine) for establishing the substrate specificity. Test samples (30S) were incubated with PNPA and various concentrations (1 mM and 5 mM) of the different substrates and thereafter the NAT like activity (in triplicate) was determined (Wang et al. 2005).

### Analysis of heavy metal ions in the blue secretion

The concentrations of the metal ions (mercury, lead and arsenic) in the blue secretion of the mollusk specimens collected during pre-monsoon (May), monsoon (August) and post-monsoon (November) were determined by atomic absorption spectrometer (AA 303, Thermo Scientific) to deduce a possible relationship between the observed NAT activity with the concentrations of the various heavy metal present in the secretion. Wet digestion method using HNO_3_/HClO_4_ was adopted for the determination of trace metals by atomic absorption spectrophotometer as described by Soares et al. ( 2000). The temperature-time combinations were optimized for each element, and the accuracy, precision, selectivity, and sensitivity were verified with reference sample. The blanks were made in the same way without using any sample. All test samples were prepared in triplicate.

### Statistical analysis

Values are represented as the mean ± SEM of the three independent experiments and statistical significance was determined using one-way analysis of variance (ANOVA) followed by Dunnett’s tests for multiple comparisons. Statistical significance was assessed using Student’s t-test was used in two-group comparisons.

## References

[CR1] Adhikari D, Samanta SK, Dutta A, Roy A, Vedasiromoni JR, Sen T (2007). *In vitro* hemolysis and lipid peroxidation-inducing activity of the tentacle extract of the sea anemone (*Paracondylactis indicus* Dave) in rat erythrocytes. Indian J Pharmacol.

[CR2] Amin B, Ismail A, Kamarudin MS, Arshad A, Yap CK (2005). Heavy Metals (Cd, Cu, Ph and Zn) Concentrations in *Telescopium telescopium* from Dumai Coastal Waters, Indonesia. Pertanika J Trop AgricSci.

[CR3] Blum M, Grant DM, McBride W, Heim M, Meyer UA (1990). Human arylamine N-acetyltransferase genes: Isolation, chromosomal localization, and functional expression. DNA Cell Biol.

[CR4] Bradford MM (1976). A rapid and sensitive method for the quantitation of microgram quantities utilizing the principle of protein dye binding. Anal Biochem.

[CR5] Brooke EW, Davies SG, Mulvaney AW, Pompeo F, Sim E, Vickers RJ (2003). An approach to identifying novel substrates of bacterial arylamine N-acetyltransferases. Bioorg Med Chem.

[CR6] Chung JG (1998). Purification and characterization of an Arylamine N-Acetyltransferase from the bacteria *Aeromonas hydrophilia*. Curr Microbiol.

[CR7] Datta U, Hembram ML, Roy S, Mukherjee P (2010). Sperm morphology and natural biomolecules from marine snail *Telescopium telescopium*: a phylogenetic perspective. Int J Morphol.

[CR8] Deguchi T, Sakamoto Y, Sasaki Y, Uyemura K (1988). Arylamine N-acetyltransferase from chicken liver. J BiolChem.

[CR9] Dellali M, Gnassia-Barelli M, Romeo M, Aissa P (2001). The use of acetylcholinesterase activity in *Ruditapes decussatus* and *Mytilus galloprovincialis* in the biomonitoring of Bizerta lagoon. Comp Biochem Physiol C.

[CR10] Escartin E, Porte C (1997). The use of cholinesterase and carboxylesterase activities from Mytilusgalloprovincialis in pollution monitoring. Environ Toxicol Chem.

[CR11] Gaitonde D, Sarkar A, Kaisary S, Silva CD, Dias C, Rao DP, Ray D, Nagarajan R, De Sousa SN, Sarker S, Patill D (2006). Acetyl cholinesterase activities in marine snail (*Cronia contracta*) as a biomarker of neurotoxic contaminants along the Goa coast, West coast of India. Ecotoxicology.

[CR12] Gaudet SJ, Slominski A, Etminan M, Pruski D, Paus R, Namboodiri MA (1993). Identification and characterization of two isozymic forms of arylamine N-acetyltransferase in Syrian hamster skin. J Invest Dermatol.

[CR13] Glenn AE, Karagianni EP, Ulndreaj F, Boukouvala S (2010). Comparative genomic and phylogenetic investigation of the xenobiotic metabolizing arylamine N-acetyltransferase enzyme family. FEBS Lett.

[CR14] Hearse DJ, Weber WW (1973). Multiple N-acetyltransferases and drug metabolism, tissue distribution, characterization and significance of mammalian N-acetyltransferase. Biochem J.

[CR15] Hui CS, Kuo HM, Yu CS, Li TM (2004). Evidence for arylamine N-acetyltransferase activity in Klebsiella pneumonia. J Microbiol Immunol Infect.

[CR16] Joseph KO, Srivastava JP (1993). Pollution of estuarine systems: heavy metal contamination in the sediments of estuarine systems around madras. J Indian Soc Soil Sci.

[CR17] Kawamura A, Graham J, Mushtaq A, Tsiftsoglou SA, Vath GM, Hanna PE, Wagner CR, Sim E (2005). Eukaryotic arylamine N-acetyltransferase. Investigation of substrate specificity by high-throughput screening. Biochem Pharmacol.

[CR18] Kumar AA, Dipu S, Sobha V (2011). Seasonal variation of heavy metals in cochin estuary and adjoining Periyar and Muvattupuzha rivers, Kerala, India. Global J Environ Res.

[CR19] Mattano SS, Land S, King CM, Weber WW (1989). Purification and biochemical characterization of hepatic arylamine N-acetyltransferase from rapid and slow acetylator mice: identity with aryl hydroxamic acid N, O-acyltransferase and N-hydroxyarylamineOacetyltransferase. Mol Pharmacol.

[CR20] Pakrashi A, Datta U, Choudhury A (1992). A search for immune contraceptive agent from marine sources–role of antispermatheca globulin of Telescopium telescopium on fertility regulation in male rat. Indian J Exp Biol.

[CR21] Pakrashi A, Roy P, Datta U (2001). Antimicrobial effect of protein(s) isolated from a marine mollusk *Telescopium telescopium*. Indian J Physiol Pharmacol.

[CR22] Pfeifer S, Doris S, Dippner JW (2005). Effect of temperature and salinity on acetyl cholinesterase activity, a common pollution biomarker, in Mytilus sp. from the south-western Baltic Sea. J Exp Mar Biol Ecol.

[CR23] Sabbagh A, Marin J, Veyssière C, Lecompte E, Boukouvala S, Poloni ES, Darlu P, Crouau-Roy B (2013). Rapid birth-and-death evolution of the xenobiotic metabolizing NAT gene family in vertebrates with evidence of adaptive selection. BMC Evol Biol.

[CR24] Samanta SK, Kumar KT, Roy A, Karmakar S, Lahiri S, Palit G, Vedasiromoni JR, Sen T (2008). An insight on the neuropharmacological activity of Telescopium telescopium–a mollusc from the Sunderban mangrove. Fundam Clin Pharmacol.

[CR25] Samanta SK, Adhikari D, Karmakar S, Dutta A, Roy A, Manisenthil KT, Roy D, Vedasiromoni JR, Sen T (2008). Pharmacological and biochemical studies on *Telescopium telescopium* – a marine mollusk from the Mangrove regions. Orient Pharm Exp Med.

[CR26] Sarkar SK, Cabral H, Chatterjee M, Cardoso I, Bhattacharya AK, Satpathy KK, Alam MA (2008). Biomonitoring of heavy metals using the bivalve molluscs in Sunderban mangrove wetland, northeast coast of Bay of Bengal (India): possible risks to human health. Clean- Soil Air Water.

[CR27] Scopes R (1982). Protein Purification: Principles and Practice.

[CR28] Sim E, Lack N, Wang CJ, Long H (2008). Arylamine N-acetyltransferases: structural and functional implications of polymorphisms. Toxicology.

[CR29] Soares ME, Bastos ML, Ferreira M (2000). Selective determination of chromium (VI) in powdered milk infant formulas by electrothermal atomization atomic absorption spectrometry after Ion exchange. J Assoc Official Agric Chem.

[CR30] Sturm A, da Silva de Assis HC, Hansen PD (1999). Cholinesterases of marine teleost fish: enzymological characterization and potential use in the monitoring of neurotoxic contamination. Mar Environ Res.

[CR31] Swadling P (1977). Central province shellfish resources and their utilization in the prehistoric past of Paua New Guinea. Veliger.

[CR32] Tsangaris C, Kormas K, Strongyloudi E, Hatzianestis I, Neofitou C, Andral B, Galgani F (2010). Multiple biomarkers of pollution effects in caged mussels on the Greek coastline. Comp Biochem Physiol C Toxicol Pharmacol.

[CR33] Vagena E, Fakis G, Boukouvala S (2008). Arylamine N-acetyltransferases in prokaryotic and eukaryotic genomes: a survey of public databases. Curr Drug Metab.

[CR34] Wang H, Vath GM, Kawamura A, Bates CA, Sim E, Hanna PE, Wagner CR (2005). Over-expression, purification, and characterization of recombinant human arylamine N-acetyltransferase 1. Protein J.

[CR35] Whitaker DP, Goosey MW (1993). Purification and properties of the enzyme arylamine N-acetyltransferase from the housefly *Musca domestica*. Biochem J.

[CR36] Yap CK, Noorhaidah A (2011). Assessment of bioavailability and contamination by Cd in the tropical intertidal area, using different soft tissues of *Telescopium telescopium*: Statistical multivariate analysis. J Sustain Sci Manage.

[CR37] Zuykov M, Pelletier E, Harper DAT (2013). Bivalve mollusks in metal pollution studies: from bioaccumulation to biomonitoring. Chemosphere.

